# Quality appraisal of gestational diabetes mellitus guidelines with AGREE II: a systematic review

**DOI:** 10.1186/s12884-019-2597-8

**Published:** 2019-12-05

**Authors:** Mengxing Zhang, Yingfeng Zhou, Jie Zhong, Kairong Wang, Yan Ding, Li Li, Xiuhong Pan

**Affiliations:** 1 JBI Evidence Based Nursing Cooperation Center, School of Nursing, Fudan University, Shanghai, China; 2 Obstetrics and Gynecology Hospital of Fudan University, Shanghai, China; 3 Shanghai Pudong Hospital, Shanghai, China

**Keywords:** Gestational diabetes mellitus (GDM), Clinical practice guidelines (CPGs), AGREE II

## Abstract

**Background:**

Several societies and associations have produced and disseminated clinical practice guidelines (CPGs) for gestational diabetes mellitus (GDM). However, the quality of such guidelines has not been appraised so far. This study aims to evaluate the quality of CPGs for GDM published in the last decade using the AGREE II instrument.

**Methods:**

A systematic search of the National Institute for Health and Care Excellence, New Zealand Guidelines Group, Scottish Intercollegiate Guidelines Network, Medlive, American Diabetes Association, Canadian Diabetes Association, International Diabetes Federation, as well as PubMed, Web of Science, Embase, China National Knowledge Infrastructure, Wanfang Chinese Periodical Database, and VIP Chinese Periodical Database was conducted from inception to June 2018. The quality was assessed by four trained researchers independently, using the AGREE IIinstrument.

**Results:**

A total of 13 guidelines, published from 2009 to 2018, were finally included. Among them, 11 guidelines were evidence-based guidelines, and 2 were expert consensus. Scores for each of the six AGREE II domains(Median ± IQR) were 94 ± 11, 89 ± 53, 58 ± 37, 100 ± 6, 79 ± 48, 100 ± 71 and 67% ± 42%, and guidelines based on expert consensus generally scored lower than evidence-based guidelines (Z = -2.201, *p* = 0.028). Overall score of 10 guidelines were 5 points and above, and four guidelines were 7 points. Among six domains, two domains: Scope and Purpose, and Clarity of Presentation, had high scores; however, the domains of Rigor of Development, Stakeholder Involvement and Editorial Independence received lower scores.

**Conclusions:**

In general, the methodological quality of GDM guidelines is high, and evidence-based guidelines are superior to expert consensus. However, the domains of Rigor of Development, Stakeholder Involvement and Editorial Independence still need improvement. A systematic approach in the development of these guidelines and updating timely is needed. In some regions, more attention for guideline adaptation is recommended.

## Background

Gestational diabetes mellitus (GDM) is defined as “any degree of glucose intolerance with onset or first recognition during pregnancy” [1]. The prevalence of GDM is increasing every year, not only because diagnostic criteria has changed and more women with high blood glucose are regarded as GDM patients on the basis of the Hyperglycemia and Adverse Pregnancy Outcome (HAPO) study [2], but also have to do with advanced maternal age, family history of diabetes, inactive physical activity, obesity, and other risky behaviors [3, 4]. GDM is gradually becoming a major concern in the field of gynecology and obstetrics. The International Diabetes Federation (IDF) estimates that approximately one in six live births (16.2%) are to women with some form of hyperglycemia in pregnancy, while the majority (85.1%) is due to GDM [5].

It is acknowledged that GDM is associated with a higher incidence of maternal and fetal morbidity, and may have long-term sequelae in offspring, leading to a higher social burden [6–8]. In 2008, a total of 25,505 pregnant women at 15 centers in nine countries were included in the HAPO study. The study primarily revealed the prevailing association of various degrees of maternal glucose intolerance with increased birth weight and increased cord-blood serum C-peptide levels, and this provided evidence on the association between maternal glycemia and adverse outcomes [2]. Neonatal complications, known as macrosomia, birth asphyxia, hyperbilirubinemia and hypoglycemia, are significantly higher in mothers with GDM than non-diabetic mothers [9]. As maternal outcomes have shown, GDM is a pathway to type 2 diabetes [10]. To provide treatment for mild GDM in addition to routine obstetric care, additional direct costs would be incurred [8, 11, 12]. In 2007, it was estimated that in the US, GDM increased national medical costs by $636 million ($596 million for maternal costs and $40 million for neonatal costs) [12]. In Australia, a multi-center randomized clinical trial revealed that, for every 100 women with a singleton pregnancy, an additional direct cost of AUD53, 985 was incurred at the obstetric hospital, and additional charges of AUD6521 were incurred by women and their families [11].

However, maternal and fetal adverse outcomes can be significantly reduced if GDM women are properly managed [13, 14]. As the most authoritative form, GDM clinical practice guidelines (CPGs) are widely distributed by professional medical associations, and clinicians rely on GDM guidelines for guidance when making decisions for patients. Nevertheless, previous studies only have shown that the quality of guidelines on the management of diabetes in pregnancy are suboptimal [15], but the critical appraisal for GDM CPGs has not been studied before. As greater attention is being placed on evidence-based medicine in the last decade, the number of guidelines based on evidence published has increased, however they lack systematic quality evaluation. Hence, the objective of this study is to assess the methodological quality of CPGs for GDM management using the AGREE II(Appraisal of Guidelines for Research and Evaluation II)instrument [16].

## Methods

### Inclusion and exclusion criteria

Two reviewers independently reviewed the guidelines yielded by the search, based on these inclusion criteria: 1) full guideline is available in English or Chinese; 2) CPG is systematically developed under the auspices of medical specialty associations, government agencies at the federal, state or local level or health care organizations; 3) CPG contains recommendations regarding GDM interventions; 4) the guideline has been developed, reviewed or revised within the last 10 years.

The following literature was excluded: 1) translations of guidelines; 2) short summaries, abstracts, brief versions or only sections of guidelines; 3) guidelines for patients and editorials.

### Data sources and searches

One reviewer performed a search of the following electronic databases from inception to June 2018: Guideline websites of the National Institute for Health and Care Excellence (NICE), New Zealand Guidelines Group (NZGG), Scottish Intercollegiate Guidelines Network (SIGN) and China Medlive; websites of medical specialty associations, such as American Diabetes Association (ADA), Canadian Diabetes Association (CDA) and International Diabetes Federation (IDF); and PubMed, Web of Science, Embase, China National Knowledge Infrastructure (CNKI), Wanfang Chinese Periodical Database and, VIP Chinese Periodical Database.

The search strategy used keywords “pregnancy”, “gravida*”, “conception”, “maternity”, “diabetes”, “hyperglycemia”, “insulin resistance”, “glucose intolerance”, “guideline”, “criteria”, “recommendation” and “standard”. For example, the PubMed search strategy is presented in Table 1.

**Table 1:** Search strategy (PubMed)

#1 pregnancy OR gravida* OR gestational OR conception OR maternity[Ti]	
#2 diabetes OR hyperglycemia* OR insulin resistance OR Glucose intolerance[Ti]	
#3 guide* OR standard* OR criteria* OR consensus [Ti]	
(#1) AND (#2) AND (#3)	

### Quality appraisal

Quality appraisal for included guidelines was conducted by four reviewers. Four trained appraisers (YF Zhou, MX Zhang, J Zhong, and KR Wang) independently evaluated GDM CPGs using the AGREE II instrument [16]. According to AGREE II handbook [16], the AGREE II consists of 23 key items categorized into six domains followed by two global rating items (“Overall Assessment”). Each of the AGREE II items and the two global rating items is rated on a seven-point scale (1–strongly disagree to 7–strongly agree), and the reviewers evaluated according to the quality and completeness of the guideline report. The six domain scores are independent and will not be aggregated into a single quality score. Domain scores are calculated by summing up the scores of the individual items in a domain and by scaling the total as a percentage of the maximum possible score for that domain: (obtained score – minimum possible score)/(maximum possible score – minimum possible score). The Consortium of AGREE II has not set minimum domain scores or patterns of scores across domains to differentiate between high quality and poor quality [16]. Thus, in this study, whether a GDM CPG is recommended is not determined by the domain scores.

### Data extraction and analysis

After quality appraisal, data extraction and analysis was performed by one reviewer and checked by another one. Any discrepancies were resolved by discussion between them or with a third party. The main characteristics of these publications were extracted, including development organization, publication year, development method, and the number of references. In addition, the results of the AGREE II appraisals (standardized domain scores, and results of the overall assessments) were extracted from the publications included and a descriptive statistics analysis undertaken.

The IBM SPSS Version 25.0 (Armonk, NY: IBM Corp) was used for all statistical analyses. Descriptive analysis values included median and inter-quartile range (IQR). Differences between CPG and expert consensus scores were calculated based on the Wilcoxon Signed Rank Test Z-score, and *p* values of 0.05 or less were considered significant. In order to measure agreement among reviewers, intra-class correlation coefficients (ICC) were also calculated. ICC values above 0.75 were considered to represent good reliability.

## Results

### Selection of relevant publications

**Fig. 1:**
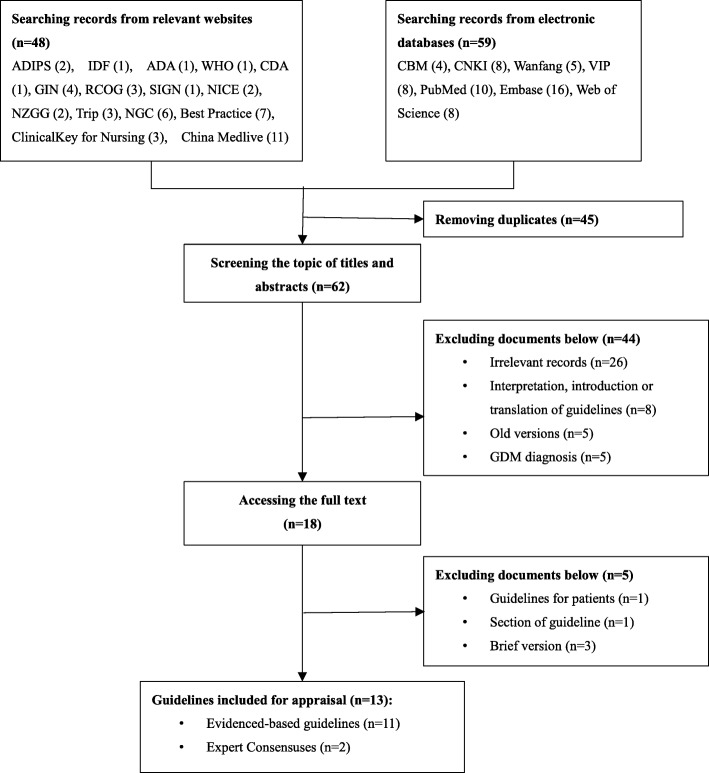
Flowchart of the systematic literature search and selection

The systematic search retrieved a total of 107 publications, including 48 searched in relevant websites and 59 searched in electronic databases. Two reviewers independently selected guidelines according to the inclusion criteria. After excluding 48 duplicated records, 59 publications were considered to be potentially relevant. Then, the publications were screened by title and abstract as well as full text. Eventually, 13 guidelines fulfilled the inclusion criteria (Fig. 1).

### Guideline characteristics

Thirteen guidelines were finally selected; these were from ADA [17], CDA [18], NICE [19], API [20], NGC [21], NZGG [22], SIGN [23], Chinese Medical Association [24] as well as IDF [25], FIGO [26], Queensland [27], HKCOG [28] and A.N.D. [29]. There was only one guideline published in 2009 [25], whereas the other 12 guidelines were published from 2013 to 2018. In terms of methodology, 11 guidelines [17–23, 25–27, 29] were developed based on evidence and two guidelines were based on expert consensus [24, 28]. The evidence-based guidelines used five kinds of grading systems, including GRADE, ADA, AACE, CDA, SIGN system, among which only four guidelines [19, 21, 22, 26] used the GRADE system (Table 2).

**Table 2:** Characteristics of the 13 guidelines

	Guidelines	Country	Development institute	Publication year	Evidence grading system	Type
1	Standards of medical care in diabetes (2018) [17]	America	ADA	2018	ADA evidence grading system	Evidence-based
2	Gestational Diabetes (2016) Evidence-Based Nutrition Practice Guideline [29]	America	A.N.D.	2016	Not mentioned	Evidence-based
3	Consensus Evidence-based Guidelines for Management of Gestational Diabetes Mellitus in India [20]	India	API	2014	AACE evidence grading system	Evidence-based
4	Clinical Practice Guidelines: Diabetes and Pregnancy [18]	Canada	CDA	2013	CDA evidence grading system	Evidence-based
5	Initiative on gestational diabetes mellitus: a pragmatic guide for diagnosis, management, and care [31]	International	FIGO	2015	GRADE	Evidence-based
6	Global Guideline on Pregnancy and Diabetes [30]	International	IDF	2009	Not mentioned	Evidence-based
7	Diabetes and Pregnancy: an Endocrine Society Clinical Practice Guideline [21]	America	Endocrine Society	2013	GRADE	Evidence-based
8	Diabetes in pregnancy: management from preconception to the postnatal period [19]	England	NICE, NCC-WCH	2015	GRADE	Evidence-based
9	Screening, Diagnosis and Management of Gestational Diabetes in New Zealand: a clinical practice guideline [22]	New Zealand	NZGG	2014	GRADE	Evidence-based
10	Queensland Clinical Guideline: Gestational diabetes mellitus [27]	Queensland	Department of Health	2015	Not mentioned	Evidence-based
11	Management of diabetes: a national clinical guideline [23]	Scotland	SIGN	2013	SIGN evidence grading system	Evidence-based
12	Diagnosis and Management of diabetes in pregnancy: a clinical practice guideline (2014) [24]	China	CMA	2014	Consensus of experts	Expert consensus
13	Guidelines for the Management of Gestational Diabetes Mellitus [28]	HKCOG	HKCOG	2016	Not mentioned	Experts consensus

### Methodologic quality assessment

#### Overall assessment

**Fig. 2:**
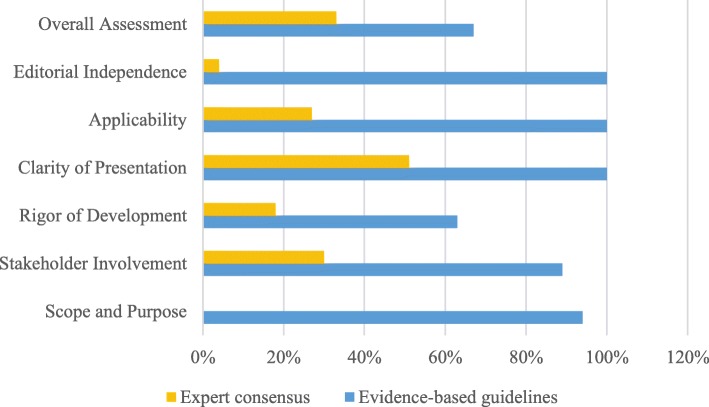
Median scores of guidelines included

The overall guideline quality varied considerably. But in general, the methodological quality of most guidelines was acceptable. Most domains in evidence-based guidelines scored more than 50%. Overall quality scores of 10 guidelines were more than 5 [17–19, 21–23, 25–27, 29], especially four guidelines [18, 19, 22, 23] of which scored 7. As to “recommendation for use”, 10 guidelines [17–19, 21–23, 25–27, 29] were rated as “A” (which means recommended). The scores of six domains were significantly different, but the domains “Scope and Purpose” and “Clarity and Presentation” consistently scored well, and the domains “Rigour of Development” and “Editorial Independence” scored relatively lower. Also, guidelines based on expert consensus generally scored lower than the evidence-based guidelines (Z = -2.201, *p* = 0.028) (Fig. 2). ICC showed that appraisers reached a high agreement (α = 0.98).

#### Domain scores

The scores of each domain are described below:

1. Scope and purpose

The overall objectives of all the evidence-based guidelines were specifically described to some degree (item 1). Seven guidelines [17, 19, 21–23, 26, 27] clearly described health question(s) covered by guidelines (item 2). The specific populations to whom the guidelines were meant to apply (item 3) were also well described in eight CPGs [17–19, 22, 23, 26, 27, 29]. However, each item of “Scope and purpose” domain was described vaguely in two expert consensuses.

2) Stakeholder Involvement

Six guidelines [17–19, 22, 23, 27] provided details of their development group members and their academic backgrounds, including individuals from all relevant professional groups, namely, diabetes specialists, obstetricians and gynecologists, methodologists, nutrition experts and diabetes specialists nurses (item 4). The remaining guidelines also mentioned multidisciplinary guideline development group but lack of details. Four guidelines [18, 19, 22, 23] sought the views and preferences of the target population, but three other guidelines [20, 24, 28] did not provide a description of the population (item 5). Eight guidelines [17–19, 21–23, 26, 29] clearly defined the target users, involving healthcare professionals, researchers, health service providers and patients (item 6).

3) Rigor of Development

The process of evidence retrieval and recommendation formation varied considerably in different guidelines. Four guidelines [18, 19, 22, 23] used a systematic search strategy (item 7), whereas three guidelines [21, 25, 26] listed recommendations and related evidence while not providing a search strategy. Three guidelines [18, 19, 22] clearly described the selection criteria (item 8) but seven guidelines did not provide these [17, 20, 21, 24–26, 28]. Six guidelines [18–23] described the strengths and limitations of evidence (item 9). Eight guidelines [17–23, 26] presented the methods for formulating the recommendations (item 10), of which four guidelines [19, 21, 22, 26] adopted the GRADE system. All guidelines except the HKCOG guideline [28] more or less considered health benefits, side effects and risks in the formulation of the recommendations (item 11). All evidence-based guidelines stated an explicit link between the recommendations and the supporting evidence (item 12). Unsurprisingly, guidelines based on expert consensus lacked descriptions of this aspect. Eight guidelines [17–19, 21, 23, 25–27] had been externally reviewed by experts prior to their publication (item 13), while another two guidelines [20, 22] did not describe whether external reviews had been conducted. Four guidelines [18, 19, 22, 23] provided a clearly procedure for updating the guideline (item 14), while four guidelines [20, 21, 25, 26] did not mentioned updating.

4) Clarity of Presentation

The recommendations of 10 guidelines [17–23, 25, 26, 29] were specific and unambiguous, while in the Queensland guideline [27] and two expert consensuses [24, 28], the important recommendations were ambiguous (item 15). All guidelines provided the alternative options for management of the condition or health issue in varying degrees (item 16). Key recommendations were easily identifiable in 11 guidelines [17–23, 25–27, 29], but they were not in the CMA [24] and HKCOG guidelines [28] (item 17).

5) Applicability

Seven guidelines [18, 19, 22, 23, 25, 26, 29] described facilitators and barriers to guideline application. On the contrary, three guidelines [17, 24, 28] did not mention these at all (item 18). Eight guidelines [17–19, 22, 23, 25–27] provided advice or tools on how recommendations can be put into practice (item 19). To be applicable, six guidelines [18, 19, 22, 23, 25, 26] took into account the potential resource implications of applying the recommendations (item 20).The ADA guideline however showed no consideration of resources. All guidelines, except the HKCOG guideline [28], presented monitoring or auditing criteria (item 21).

6) Editorial Independence

Nine guidelines [17–19, 21–23, 25–27] clarified that the views of the funding body had no influence on the contents of the guideline, and the contents of three guidelines [20, 24, 29] had no commercial interest involved although the conflict of interest had not been stated. However, the HKCOG guideline [28] did not mention conflict of interest at all (item 22). Seven guidelines [17–19, 21, 23, 26, 27] recorded and addressed the competing interests of guideline development group members, while four guidelines [20, 24, 25, 28] did not. The scores of all six domains are presented in Table 3.

**Table 3:** AGREE II domain scores

Guidelines	Scope and Purpose	Stakeholder Involvement	Rigor of Development	Clarity of Presentation	Applicability	Editorial Independence	Overall Assessment
ADA [17]	100%	89%	58%	100%	31%	100%	75%
A.N.D. [29]	94%	89%	51%	94%	77%	29%	58%
API [20]	78%	19%	58%	100%	52%	8%	50%
CDA [18]	94%	100%	100%	100%	100%	100%	100%
FIGO [26]	100%	81%	55%	100%	100%	100%	67%
IDF [25]	89%	47%	45%	100%	100%	50%	67%
NGC [21]	92%	81%	63%	100%	71%	100%	67%
NICE [19]	100%	100%	100%	100%	100%	100%	100%
NZGG [22]	100%	100%	88%	100%	100%	96%	100%
Queensland [27]	94%	92%	63%	89%	79%	100%	58%
SIGN [23]	100%	100%	97%	100%	100%	100%	100%
CMA [24]	58%	25%	25%	44%	52%	8%	33%
HKCOG [28]	56%	35%	11%	58%	2%	0%	33%
Median ± IQR	94% ± 11%	89% ± 53%	58% ± 37%	100% ± 6%	79% ± 48%	100 ± 71%	67% ± 42%
ICC	0.85	0.96	0.96	0.85	0.98	0.98	0.98

## Discussion

### Overall quality of GDM CPGs

Clinical Practice Guidelines are “statements that include recommendations intended to optimize patient care that are informed by a systematic review of evidence and an assessment of the benefits and harms of alternative care options” [30]. Therefore, CPGs have the potential to influence the care delivered by a large number of healthcare providers and consequently the outcomes for patients, which is why we should emphasize the quality of CPGs [31]. This article seeks to provide an overview and appraisal of the methodological quality of clinical guidelines on the management of GDM. The overall quality of 13 CPGs varied but scored well into some extent. The scores of seven guidelines [18, 19, 21–23, 26, 27] exceeded 50% in each domain, and the overall scores of 11 guidelines [17–23, 25–27, 29] were more than 50%, of which four guidelines [18, 19, 22, 23] scored 7 in overall quality, indicating development methodology of these guidelines was credible. This was probably because most of the guidelines included in this study were based on evidence, and the publication date was generally within the last 5 years. Recently, as a result of the development of evidence-based practice, improvements in guideline methodology, specifications for guideline formulation as well as standardization in the reporting of guidelines, the rigor of guideline development has significantly improved over time [32]. It is acknowledged that rigor of methodology is a scientific underpinning of evidence-based guidelines. Without explicit descriptions of how the available evidence is identified and selected, it is hard to ensure valid and reliable evidence-based recommendations, which decreases the quality of guideline [33]. Nevertheless, critical appraisal of the six domains reported a great deal of variability in quality, with the domains “Editorial Independence”, “Stakeholder Involvement” and “Rigor of Development” having lower scores, which was in line with the appraisal results of guidelines on diabetes management in pregnancy glycemic control performed by Marjolein in 2012 [15]. This can be explained by the fact that the score on the AGREE II instrument does not only depend on the methodological quality of the guideline, but also on the reporting quality. It may be a common issue that some essential parts are always missing both in GDM CPGs and CPGs on the management of diabetes in pregnancy.

In addition, views and preferences of the target population, competing interests of guideline development group members and procedures for updating the guideline were also influential factors in guideline quality, with only four guidelines [18, 19, 22, 23] having clear descriptions of the views and preferences of patients. Importantly, extensive clinical experience suggests that self-management by women and close cooperation between women and health care professionals play crucial roles in GDM management, so without knowing patients’ preferences, the implementation of guidelines will be definitely influenced. Similarly, to decrease the bias of the recommendations, conflict of interest disclosure was also notably missing. Four guidelines [20, 24, 25, 28] had no disclosure, indicating that recommendations may have had financial ties, which compromised the reliability of the guidelines. A case study analysis of guidelines from the Canadian Medical Association InfoBase revealed that financial ties are common among guideline authors, committee members, and drug companies that manufacture medications recommended in guidelines [34]. Thus, it is crucial that attention to the risk of bias resulting from conflicts of interest should be a priority for guideline development groups. Additionally, similar to what was revealed in other guideline appraisals, descriptions of the updating procedures of the guidelines were poor [35]. As a general rule, guidelines should be reassessed for validity every 3.6 years [36]. The WHO guideline handbook indicated that although the maximum duration of the validity of recommendations has not been set, guidelines should be updated within a minimum period of 2 years, and the maximum period of 5 years [37]. Therefore, updating guidelines in a timely manner is extremely important.

### Guideline adaptation could be an alternative

With evidence-based medicine being a critical scientific methodology in the development of CPGs, there is a growing international tendency to develop guidelines based on evidence. In this study, we included 13 guidelines, of which 11 [17–23, 25–27, 29] were evidence-based guidelines. However, in some countries or regions, there was only GDM guideline developed through expert consensus [24, 28]. Although the local context was considered, the process of the guideline development was far from being rigorous. Based on AGREE II, two expert consensus received low scores, generally between 4 and 57%, and four domains scored less than 50%, especially in the “Editorial Independence” and “Rigor of Development” domains. As a matter of fact, clinical staffs always regard guidelines as valuable and trustworthy, and they are more likely to make decisions in accordance with recommendations based on a systematic review of the evidence [38, 39]. So evidence-based guidelines are essentially needed. It is acknowledged, however, that the process of guideline development to produce quality recommendations for care, which requires an extensive search, synthesis of primary research data, objective evidence quality assessment and scientific recommendation formulation, is a resource intensive method of [40]. In addition, recommendations based on the same evidence may also differ in different countries because of cultural diversity and legislation differences [41], hence guideline translation is not proposed as an alternative approach to de novo guideline development [33, 41]. To avoid the incorrect application of guidelines and to reduce duplication of efforts while ensuring consideration of local contextual factors, guideline adaptation may be another way to integrate high quality evidence and local policies and health service resources.

### Limitations

The search for relevant publications was limited to Chinese and English language publications, and potentially relevant publications in other languages were not considered, which might lead to bias and scope limitations. Besides, the management recommendations for GDM could be presented not only in GDM CPGs, but also any other guidelines related to diabetes, so the search strategy in this research may be not comprehensive to include all relevant guidelines. Also, we may have missed guidelines published in other forms such as books and internal reports.

In terms of the appraisal instrument, the AGREE II instrument can only assess the methodological quality of the guideline but not the quality of the content of the guideline [25, 26, 30]. In addition, the domain “Rigor of development” should be considered as an important evaluation basis in evidence-based guideline appraisal. However, according to AGREE II instrument, each domain gets equal importance, which may be improper to determine the guideline quality [31].

## Conclusions

In general, the methodological quality of GDM guidelines is high, and evidence-based guidelines are superior to expert consensus. Although the development methodology of these guidelines was credible, the domains of Rigor of Development, Stakeholder Involvement and Editorial Independence still need improvement. A systematic approach in the development of these guidelines and updating guidelines in a timely manner is recommended.

However, in some countries and regions, there were only GDM guidelines developed through expert consensus, and the process of the guideline development was far from being rigorous. Given the thought of using high quality evidence and reducing duplication of efforts, guideline adaptation could be an alternative. In additional, it is crucial that clinical staffs should use guidelines on the basis of local contextual factors.

## Data Availability

All data analyzed during this study are included in this published article.

## Abbreviations

A.N.D: Academy of Nutrition and Dietetics
AACE: The American Association of Clinical Endocrinologists
ADA: American Diabetes Association
API: The Association of Physicians of India
CDA: Canadian Diabetes Association
FIGO: The International Federation of Gynecology and Obstetrics
IDF: International Diabetes Federation
NCC-WCH: National Collaborating Centre for Women's and Children's Health
NGC: National Guideline Clearinghouse
NICE: The National Institute for Health and Care Excellence
NZGG: New Zealand Guidelines Group
SIGN: Scottish Intercollegiate Guidelines Network
WHO: World Health Organization
